# Adjuvant Immunotherapy for Resectable Non-Small Cell Lung Cancer: Current Advances and Future Perspectives

**DOI:** 10.3390/cancers17132099

**Published:** 2025-06-23

**Authors:** Alice Ravasin, Lavinia Gatteschi, Valentina Massa, Mauro Iannopollo, Luca Voltolini, Alessandro Gonfiotti

**Affiliations:** 1 Thoracic Surgery Unit, Careggi University Hospital, 50134 Florence, Italy; 2 Oncology Unit, San Jacopo Hospital, Oncology Department, Central Tuscany AUSL, 51100 Pistoia, Italy; 3 Department of Experimental and Clinical Medicine, University of Florence, 50134 Florence, Italy

**Keywords:** resectable non-small cell lung cancer, adjuvant immunotherapy, immune-checkpoint inhibitors, adjuvant chemotherapy

## Abstract

Adjuvant treatment for resectable non-small cell lung cancer (NSCLC) has seen significant advancements following the introduction of immune checkpoint inhibitors (ICIs). These therapies, which enhance the immune system’s ability to recognize and target cancer cells, have demonstrated substantial improvements in disease-free survival (DFS) following surgical resection. Recent studies have shown that ICIs can extend DFS, particularly for patients with high Programmed Death-Ligand 1 (PD-L1) expression but also for those with lower levels of PD-L1, suggesting a broader potential for their application. In the IMpower010 trial, atezolizumab improved DFS compared to best supportive care (BSC) in resected stage II–IIIA NSCLC, with a hazard ratio (HR) of 0.66 (95% CI 0.50–0.88) for patients with PD-L1 expression ≥1% and 0.79 (95% CI 0.64–0.96) for the overall stage II–IIIA population. In the PEARLS/KEYNOTE-091 trial, pembrolizumab also demonstrated a DFS benefit over a placebo for patients with stage IB–IIIA disease (HR 0.76; 95% CI 0.63–0.91), with a median DFS of 53.6 months versus 42.0 months. Despite these promising results, challenges remain regarding the optimal selection of patients, particularly in identifying the most effective biomarkers and determining the ideal duration of treatment. While ICIs are generally well-tolerated, immune-related adverse events, although manageable, require careful monitoring, especially when ICIs are used in combination with chemotherapy. Ongoing research is focused on optimizing treatment duration and exploring combination therapies, with the objective of further improving long-term survival outcomes. The integration of immunotherapy in the adjuvant setting represents a significant advancement in the management of resectable NSCLC. This review aims to provide an overview of the current evidence supporting the use of ICIs in the adjuvant treatment of NSCLC, focusing on treatment efficacy, safety profiles, and ongoing research into biomarkers and combination therapies.

## Simple Summary

Non-small cell lung cancer (NSCLC) continues to be a leading cause of cancer-related mortality worldwide, even in the early stages. Despite significant advances in treatment strategies, prognosis for patients with resectable NSCLC remains challenging, mainly due to the high risk of recurrence. Adjuvant immunotherapy has emerged as a promising approach to improving outcomes, particularly by reducing recurrence rates and enhancing survival among patients following surgical resection. This review explores the current challenges in managing resectable NSCLC, focusing on critical aspects such as patient selection, optimal treatment regimens, and potential side effects.

## 1. Introduction

### 1.1. Background

Lung cancer remains the leading cause of cancer-related mortality worldwide, with non-small cell lung cancer (NSCLC) accounting for approximately 80–85% of all diagnosed cases [1]. In 2022, it was estimated that lung cancer caused over 1.8 million deaths globally, representing nearly 18% of all cancer-related deaths [2]. The incidence and mortality rates continue to rise, particularly in low- and middle-income countries, where access to early detection and treatment remains limited [3].

Despite significant advancements in diagnostic and therapeutic strategies, NSCLC continues to pose substantial challenges, particularly in early-stage disease. Approximately 50% of NSCLC patients present with localized or locally advanced disease upon diagnosis [4]. While surgical resection is the cornerstone of treatment for early-stage NSCLC, the recurrence rates, particularly in stage II–III patients, highlight the limitations of current therapeutic approaches. The five-year survival rates decrease significantly with an increase in the stage, with 60% of patients with stage II disease surviving five years, which drops to 24% in stage IIIB, despite curative-intent surgery [5]. The recurrence of disease, often due to micrometastases that are not detectable at the time of resection, continues to be a major concern [6,7].

In recent years, targeted therapies and immune checkpoint inhibitors (ICIs) have revolutionized the treatment landscape, particularly for metastatic disease. ICIs such as nivolumab, pembrolizumab, atezolizumab, and durvalumab have demonstrated remarkable survival benefits, leading to their adoption as first-line therapies for patients with advanced metastatic NSCLC [8,9,10,11]. The success of ICIs in the metastatic setting has spurred increasing interest in applying these therapies at earlier stages of the disease. Several clinical trials have explored the use of ICI-based therapies in the neoadjuvant (preoperative), perioperative [12], and adjuvant (postoperative) settings [4]. ICI-based therapies have shown promising results suggesting potential improvements in survival outcomes compared to chemotherapy alone. Additionally, targeted therapies such as osimertinib for patients with epidermal growth factor receptor (EGFR) mutations have further advanced the field, marking a significant shift toward personalized medicine in the management of early-stage NSCLC [13,14].

Despite these promising developments, challenges remain in defining optimal treatment strategies for early-stage NSCLC. The lack of direct comparative trials between neoadjuvant ICI therapy and the traditional approach of surgery followed by adjuvant chemotherapy creates uncertainty in clinical decision-making.

### 1.2. The Rationale of Adjuvant Immunotherapy for NSCLC

Surgical resection remains the primary treatment for early-stage NSCLC [15], yet the postoperative period presents significant immunological challenges. The process of tumor removal induces a transient “window of immunosuppression” in the host, which can last anywhere from several days to weeks [16]. During this time, the immune system’s ability to detect and eliminate residual cancer cells is profoundly impaired, providing a window for occult tumor cells to proliferate and evade immune surveillance [17]. This immunosuppressive state in the postoperative microenvironment has prompted increasing interest in the potential of adjuvant immunotherapy, particularly ICIs, to improve outcomes for NSCLC patients following surgical resection.

ICIs, which target pathways such as the Programmed Cell Death Protein 1 (PD-1) or its ligand (PD-L1) and Cytotoxic T-Lymphocyte Antigen 4 (CTLA-4), have revolutionized the treatment of metastatic NSCLC.

ICIs’ success in the metastatic setting has spurred investigations into their use in earlier stages of disease, particularly in the adjuvant setting following surgical resection. The hypothesis driving these efforts is that ICIs can overcome the immunosuppressive effects of the postoperative microenvironment, thereby enhancing the immune system’s ability to recognize and target residual tumor cells, including micrometastases, that may have been disseminated during surgery [13,18]. This approach is intended to prevent recurrence and improve long-term survival outcomes.

In the context of postoperative NSCLC management, ICIs are being explored not only as monotherapies but also in combination with adjuvant chemotherapy. Chemotherapy remains a standard treatment following surgery, particularly with respect to platinum-based regimens, which have led to modest improvements in overall survival (OS) [19,20]. In patients with early-stage NSCLC, adjuvant chemotherapy has been associated with a 5% increase in cure rates [21], and regimens such as platinum and vinorelbine have been shown to improve outcomes by 10–15% in stage II and III disease [22]. While chemotherapy improves survival, it also comes with substantial toxicity, and its effectiveness is limited, emphasizing the need for more targeted therapeutic approaches.

The rationale for combining ICIs with chemotherapy lies in their complementary mechanisms. Chemotherapy can lead to the destruction of tumor cells, increasing the release of neoantigens that can trigger immune responses. This process may expose new targets for the immune system, which ICIs can then help recognize and eliminate. By combining chemotherapy with ICIs, it may be possible to enhance both the immune response and the cytotoxic effects on the tumor, thereby maximizing therapeutic efficacy while potentially reducing the risk of recurrence.

The immunosuppressive nature of the post-surgical tumor microenvironment is one of the key factors contributing to recurrence and metastasis. After surgery, the body undergoes various stress responses, including changes in cytokine levels, growth factors, and clotting factors [23]. These alterations lead to increased infiltration of regulatory immune cells, upregulation of immune checkpoints like PD-1, and decreased T-cell proliferation, all of which hinder the immune system’s ability to respond effectively to residual tumor cells. Additionally, impaired natural killer (NK) cell cytotoxicity further contributes to a compromised anti-tumor immune response [24,25]. Therefore, the use of ICIs in the adjuvant setting is intended to reverse or mitigate these effects, potentially reducing the risk of metastatic growth and recurrence.

Incorporating ICIs into the postoperative treatment regimen for NSCLC holds significant promise, particularly as their favorable toxicity profiles compared to traditional chemotherapy offer a more manageable treatment option. Moreover, the growing understanding of the immune-related changes in the tumor microenvironment suggests that modulating these changes with ICIs could be as crucial in early-stage disease as it is in metastatic cancer [26]. In the adjuvant setting, ICIs may not only enhance immune responses but also improve the immune system’s ability to recognize and target residual tumor cells, providing a crucial advantage over traditional chemotherapy alone [27].

This review aims to examine the current state of adjuvant immunotherapy in early-stage resectable NSCLC, discussing emerging clinical data, the rationale behind its use, and the future directions for incorporating ICIs into treatment regimens aimed at improving long-term survival outcomes.

## 2. Materials and Methods

A comprehensive literature search was conducted to identify English-language articles on adjuvant immunotherapy for early-stage NSCLC using PubMed with the keywords “adjuvant immunotherapy”, “early-stage NSCLC”, and “resectable lung cancer”.

This search identified 269 articles. Based on relevance, recency, and clinical significance, a total of 122 of these articles were deemed eligible.

Additionally, ClinicalTrials.gov was searched with the term “adjuvant immunotherapy in NSCLC” to identify relevant clinical trials. Inclusion criteria encompassed peer-reviewed studies focusing on adjuvant immunotherapy applied to patients with early-stage, resectable NSCLC that reported clinical outcomes such as survival rates, response rates, or recurrence-free survival. Studies were excluded if they were not original research articles, involved metastatic or unresectable NSCLC, or lacked sufficient data on clinical outcomes. The data extracted included study design, patient demographics, immunotherapy regimens, and reported outcomes. A qualitative synthesis was performed to summarize the efficacy and safety profiles of adjuvant immunotherapy strategies in early-stage NSCLC. As this study analyzed existing published data, no new ethical approval was required.

## 3. Results

### 3.1. Adjuvant Setting

#### 3.1.1. PD-L1 Inhibitors

The role of anti-PD-L1 agents in the adjuvant treatment of early-stage NSCLC has been primarily investigated through the IMpower010 trial (NCT02486718), which evaluated the efficacy of atezolizumab. This phase III trial enrolled 1280 patients with completely resected stage IB–IIIA NSCLC; among them, 1005 who had already received platinum-based adjuvant chemotherapy were randomized to receive either atezolizumab or best supportive care (BSC). This study was designed with a hierarchical analysis plan, starting with patients with PD-L1 Tumor cell (TC) expression ≥1% with stages II–IIIA, followed by all stage II–IIIA patients regardless of PD-L1 status, and finally the full intention-to-treat (ITT) population, which included stage IB–IIIA patients.

For patients with PD-L1 TC ≥ 1% and stage II–IIIA disease, atezolizumab demonstrated a significant DFS benefit compared to BSC (HR 0.66; 95% CI 0.50–0.88; *p* = 0.0039). The median DFS was not reached in the atezolizumab arm versus 35.3 months in the BSC group. A modest DFS benefit was also observed in the overall stage II–IIIA population, with a median DFS of 42.3 months in the atezolizumab arm compared to 35.3 months with BSC (HR 0.79; 95% CI 0.64–0.96; *p* = 0.020). However, in the ITT population including patients with stage IB–IIIA disease, the improvement did not reach strong statistical significance (HR 0.81; 95% CI 0.67–0.99; *p* = 0.040). Notably, patients with high PD-L1 expression (≥50%) derived the greatest benefit (HR 0.43; 95% CI 0.27–0.68).

Subgroup analyses from IMpower010 revealed consistent benefits in Asian patients with PD-L1 TC ≥1%, while patients harboring EGFR mutations or anaplastic lymphoma kinase (ALK) rearrangements did not appear to benefit from atezolizumab, suggesting limited efficacy of PD-L1 blockade in these molecular subtypes. The safety profile of atezolizumab was generally manageable, with the most common adverse events being hypothyroidism (11%), pruritus (9%), and rashes (8%), while serious treatment-related adverse events were reported for 7% of patients [28]. DFS was the primary endpoint of this study, and key secondary endpoints included OS and long-term DFS at 3 and 5 years [29]. The trial is ongoing and it has provided foundational support for the application of adjuvant immunotherapy to patients with high PD-L1 expression [30].

Other anti-PD-L1 agents are also under investigation in the adjuvant setting. The BR31 trial (NCT02273375) evaluated the impact of durvalumab on the DFS of stage IB–IIIA NSCLC patients following surgery. Patients were randomized to receive durvalumab or placebo for one year, with the primary endpoint being DFS for those with PD-L1 expression ≥25%. The study also included patients without EGFR mutations or ALK rearrangements [31].

The MERMAID-1 trial (NCT04385368) focuses on patients with stage II–III NSCLC and minimal residual disease (MRD) detected via circulating tumor DNA (ctDNA) following surgery. This trial evaluates whether adjuvant durvalumab improves DFS among MRD-positive patients [32]. Another related study, MERMAID-2 (NCT04642469), extends this investigation by testing the benefit of durvalumab for up to 24 months in patients who have become MRD-positive during surveillance after surgery [33]. Both MERMAID trials aim to explore the potential utility of ctDNA in guiding adjuvant treatment.

These studies represent a shift toward biomarker-driven strategies that aim to personalize adjuvant immunotherapy and optimize benefits by targeting minimal residual disease.

#### 3.1.2. PD-1 Inhibitors

Pembrolizumab, an anti-PD-1 agent, has been evaluated in the PEARLS/KEYNOTE-091 trial (NCT02504372), a phase III randomized study including 1177 patients with resected stage IB–IIIA NSCLC. The participants were randomized to receive either pembrolizumab or a placebo following surgery, with adjuvant chemotherapy being optional and administered at the treating physician’s discretion. The primary endpoint was DFS, with OS designated as a key secondary endpoint.

This trial demonstrated that pembrolizumab significantly prolonged DFS compared to a placebo, with a median DFS of 53.6 months versus 42.0 months, respectively (HR 0.76; 95% CI 0.63–0.91; *p* = 0.0014). Interestingly, in the PD-L1 tumor proportion score (TPS) ≥50% subgroup, the DFS difference did not reach statistical significance (HR 0.82; 95% CI 0.57–1.18; *p* = 0.14), highlighting potential variability in predictive biomarker performance across different ICIs. Subgroup analyses indicated a greater benefit for current smokers, patients with EGFR alterations, and those with a non-squamous histology. Notably, patients who had received prior adjuvant chemotherapy derived more pronounced benefits (HR 0.73; 95% CI 0.60–0.89) compared to those who did not receive chemotherapy (HR 1.25; 95% CI 0.76–2.05) [34].

The safety profile of pembrolizumab was manageable, although grade 3 or worse adverse events occurred in 34% of treated patients, compared to 26% in the placebo arm [35]. This trial supports the use of pembrolizumab in the adjuvant setting, particularly for patients who are receiving chemotherapy, but further research is needed to better understand its effects on subgroups such as those with a squamous histology or who have not received adjuvant chemotherapy. Based on these findings, pembrolizumab was approved by the FDA on 26 January 2023, for use as an adjuvant therapy following surgery and platinum-based chemotherapy for patients with stage IB–IIIA NSCLC, regardless of PD-L1 expression [36]. Further exploration of pembrolizumab’s role is ongoing in the ACCIO trial (NCT04267848), which examines sequential and concurrent administration of pembrolizumab with chemotherapy over 17 cycles [37].

Meanwhile, nivolumab is being evaluated in the ANVIL trial (NCT02595944), part of the ALCHEMIST platform, which targets patients with resected NSCLC who are not eligible for EGFR or ALK-directed therapies. This trial aims to assess the drug’s impact on both DFS and OS, with the goal of improving survival by 30% and 33%, respectively [38]. Additionally, the NADIM-ADJUVANT trial (NCT04564157) investigates nivolumab in combination with chemotherapy administered concurrently and sequentially in stage IB–IIIA disease [39]. The LungMate-008 trial (NCT04772287) assesses toripalimab, a novel anti-PD-1 agent, in EGFR/ALK-negative patients following adjuvant chemotherapy in comparison to a placebo.

#### 3.1.3. Differences and Similarities Between IMpower010 and PEARLS/KEYNOTE-091

The PEARLS/KEYNOTE-091 and IMpower010 trials, both evaluating adjuvant ICIs in resected stage IB–IIIA NSCLC, demonstrated a significant DFS benefit brought about by pembrolizumab and atezolizumab, respectively. However, key differences were noted in terms of study design (placebo-controlled protocols in PEARLS vs. an observation-only scheme in IMpower-010) and patient characteristics. IMpower010 required mandatory cisplatin-based chemotherapy, while PEARLS/KEYNOTE-091 allowed chemotherapy at the physician’s discretion, resulting in 14% of patients not receiving any form of chemotherapy. Neither trial excluded patients with EGFR mutation- or ALK-rearrangement-positive NSCLC. Additionally, IMpower010 included a higher proportion of patients with EGFR mutations (11.6% vs. 6.2%) and ALK rearrangements (7% vs. 1.2%), whereas PEARLS/KEYNOTE-091 included fewer patients with these genetic alterations. There were also differences in PD-L1 expression in the two studies: IMpower010 found a significant correlation between PD-L1 expression (≥50%) and improved DFS, suggesting that PD-L1 is a potential biomarker for atezolizumab efficacy, while PEARLS/KEYNOTE-091 did not observe the same correlation, with pembrolizumab showing a trend toward a benefit even in PD-L1-negative patients [28,35,40]. These differences in chemotherapy regimens, the prevalence of EGFR/ALK mutations, and PD-L1 status highlight the need for further research in order to refine patient selection and better explain the long-term safety and efficacy of adjuvant ICIs in NSCLC [Table 1].

**Table 1:** IMpower010 and PEARLS/KEYNOTE-091 trial characteristics.

Clinical Trial (NCT Identifier)	Phase	Number of Patients	NSCLC Stage	Adjuvant CT	ICI	Treatment Arms	Endpoints
IMpower010 (NCT02486718)	III	1005	IB (≥4 cm)–IIIA, EGFR/ALK included	Required	Atezolizumab	Atezolizumab × 1 year vs. BSC	Primary: DFS Secondary: OS, DFS at 3 and 5 years
PEARLS/ KEYNOTE-091 (NCT02504372)	III	1177	IB (≥ 4 cm)–IIIA, EGFR/ALK included	Optional	Pembrolizumab	Pembrolizumab × 1 year vs. placebo	Primary: DFS Secondary: OS

NSCLC: non-small cell lung cancer; CT: chemotherapy; ICIs: immune checkpoint inhibitors; EGFR: epidermal growth factor receptor; ALK: anaplastic lymphoma kinase; BSC: best supportive care; DFS: disease-free survival; OS: overall survival.

#### 3.1.4. Other Checkpoint Inhibitors

Several clinical trials are exploring innovative immunotherapeutic strategies for treating early-stage NSCLC. The CANOPY-A trial (NCT03447769) evaluated the anti-Interleukin-1 beta (IL-1β) monoclonal antibody canakinumab post-chemotherapy in patients with stage II–IIIB NSCLC, aiming to exploit inflammatory pathways as therapeutic targets. Following the primary analysis, the overall benefit–risk assessment led to the decision to terminate the study. Importantly, no new safety concerns related to canakinumab were observed [41,42] [Table 2].

**Table 2:** ICIs clinical trials in the adjuvant setting.

Clinical Trial (NCT Identifier)	Phase	Number of Patients	NSCLC Stage	Adjuvant Chemotherapy	ICI	Treatment Arms	Endpoints
ACCIO (NCT04267848)	III	1210	II–IIIB	Required	Pembrolizumab	CT + concomitant pembrolizumab, then pembrolizumab vs. CT + sequential pembrolizumab vs. CT	Primary Endpoint: DFS
ANVIL (NCT02595944)	III	903	IB (≥4 cm)–IIIA	Optional	Nivolumab	Nivolumab × 1 year vs. observation	Primary Endpoint: DFS, OS
BR31 (NCT02273375)	III	1415	IB (≥4 cm)–IIIA	Optional	Durvalumab	Durvalumab vs. placebo × 1 year	Primary Endpoint: DFS
MERMAID-1 (NCT04385368)	III	332	II–III	Required	Durvalumab	CT + durvalumab/placebo	Primary Endpoint: DFS
MERMAID-2 (NCT04642469)	III	284 (ctDNA+)	II–III	Required	Durvalumab	Durvalumab vs. placebo × 2 year	Primary Endpoint: DFS in PD-L1 TC ≥1%
NADIM-ADJUVANT (NCT04564157)	III	210	IB–IIIA	Required	Nivolumab	CT + nivolumab then nivolumab vs. CT	Primary Endpoint: DFS
CANOPY-A (NCT03447769)	III	1382	II–IIIB	Required	Canakinumab	Canakinumab vs. placebo × 1 year	Primary Endpoint: DFS
LungMate-008 (NCT04772287)	III	341	II–IIIB N2	Required	Toripalimab	Toripalimab vs. placebo	Primary Endpoint: DFS

PD-L1: Programmed Death-Ligand 1; TC: tumor cell; ctDNA: circulating tumor DNA.

Emerging combination strategies are also under investigation. Other innovative therapies include treatment with QL1706, a dual-function antibody targeting both CTLA-4 and PD-1, currently being studied in the DUBHE-L-304 (NCT05487391) phase III trial, which also assesses its combination with chemotherapy in resected stage II–IIIB NSCLC patients [43].

**Figure 1:**
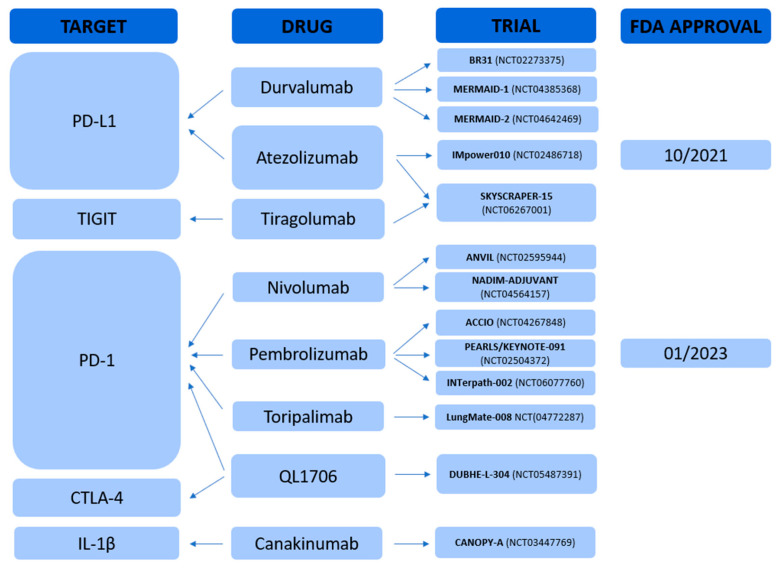
Adjuvant immunotherapy strategies for NSCLC patients. Note: PD-L1: Programmed Death-Ligand 1; TIGIT: T-cell immunoreceptor with Ig and ITIM domains; PD-1: Programmed Cell Death Protein 1; CTLA-4: Cytotoxic T-Lymphocyte Antigen 4; IL-1β: Interleukin-1 beta; FDA: Food and Drug Administration.

Similarly, the SKYSCRAPER-15 trial (NCT06267001) is evaluating the efficacy of tiragolumab, an anti–T-cell immunoreceptor with Ig and ITIM domains (TIGIT), in combination with atezolizumab. Finally, the INTerpath-002 trial (NCT06077760) is investigating V940 (mRNA-4157), a personalized neoantigen-based mRNA vaccine, in combination with pembrolizumab, reflecting a growing interest in tailored immunotherapeutic approaches [Figure 1].

### 3.2. Circulating Tumor DNA 

Circulating tumor DNA has emerged as a promising biomarker in the management of NSCLC, particularly in the context of adjuvant immunotherapy. ctDNA consists of small fragments of tumor-derived DNA that are released into the bloodstream, providing a minimally invasive method for monitoring tumor dynamics. In NSCLC, ctDNA analysis has shown significant potential in assessing MRD post-surgery [44], identifying patients at high risk of recurrence, and tracking treatment responses to adjuvant therapies [45]. Emerging evidence suggests that ctDNA can detect early signs of a relapse before conventional imaging or clinical symptoms become apparent, thus serving as a valuable tool for guiding therapeutic decisions [46].

Furthermore, quantification of ctDNA has been associated with prognosis, with elevated levels often correlating with poorer outcomes, highlighting its clinical relevance in precision oncology. A retrospective study conducted by Wang and colleagues showed that ctDNA levels can predict objective remission in NSCLC patients treated with immunotherapy, with levels lower than 3.72 ng/µL associated with favorable outcomes [47].

In the context of adjuvant immunotherapy, ctDNA detection could play a key role in refining patient selection, allowing for the identification of individuals who are most likely to benefit from additional treatment. For example, the exploratory analysis in the IMpower010 study, which investigated atezolizumab use in resected NSCLC patients, confirmed the prognostic value of ctDNA, although its predictive utility requires further validation [48].

This molecular monitoring approach holds potential for guiding the duration of immunotherapy, ensuring that patients receive the optimal treatment based on their response. However, despite the promising evidence, the routine clinical application of ctDNA still necessitates further validation through large, prospective multicenter trials to solidify its predictive and prognostic capabilities, with studies such as CheckMate 816 [49] and ADAURA [50,51] providing valuable insights into its role in shaping future therapeutic strategies.

## 4. Discussion

The integration of ICIs into the adjuvant treatment of resectable NSCLC represents a pivotal development in the management of this disease. While platinum-based chemotherapy has been the cornerstone of adjuvant therapy for decades, the emergence of immunotherapy has the potential to significantly alter treatment paradigms. Despite the promising results from recent trials, several key issues remain to be addressed in order to optimize the use of ICIs in this setting.

One of the most compelling aspects of ICIs in the treatment of resectable NSCLC is their ability to enhance the immune system’s response to cancer cells by targeting the PD-1/PD-L1 pathway. Studies such as IMpower010 and PEARLS/KEYNOTE-091 have provided substantial evidence of the efficacy of atezolizumab and pembrolizumab in improving DFS for patients with stage II-IIIA NSCLC following adjuvant chemotherapy. Notably, these trials have demonstrated that PD-L1 expression is a significant predictor of response, with patients exhibiting PD-L1 expression ≥50% experiencing the most pronounced benefits. However, both trials also showed that ICIs are effective even in patients with low or absent PD-L1 expression, suggesting that other factors, such as tumor mutational burden (TMB), may contribute to the observed outcomes [52]. This challenges the reliance on PD-L1 as the sole biomarker for treatment selection and underscores the need for a more comprehensive approach to patient stratification.

The role of TMB as a potential predictive biomarker has been gaining increasing attention in this context. A higher TMB has been associated with a greater likelihood of response to ICIs, yet its integration into clinical practice remains limited due to ongoing questions regarding its standardization and clinical utility. The absence of a universally accepted threshold for TMB further complicates its use, and additional research is required to establish its predictive value alongside PD-L1 expression. Furthermore, other emerging biomarkers, such as microsatellite instability (MSI), may offer additional insights into patient selection, but these are still in the early stages of investigation [53]. As the field moves forward, a more nuanced understanding of molecular signatures and their roles in predicting treatment response will be essential for optimizing adjuvant immunotherapy in NSCLC.

Another significant area of ongoing research is the optimal duration of adjuvant immunotherapy. Most current protocols follow a fixed one-year treatment period, but the potential benefits of extended therapy remain largely unexplored. The ADAURA trial, which investigated the use of osimertinib to treat EGFR-mutant NSCLC, demonstrated that extending therapy for up to three years significantly reduced the risk of relapse, suggesting that prolonged treatment may confer similar benefits in the immunotherapy setting [54]. While it is plausible that longer courses of ICIs could improve long-term outcomes, the risks associated with extended treatment, particularly immune-related adverse events (irAEs), must be carefully weighed. The long-term safety data for ICIs in the adjuvant setting, especially in combination with chemotherapy, is still limited, and the potential for severe irAEs could influence the overall benefit–risk profile.

The management of irAEs is another critical issue that requires further attention. Although the safety profile of ICIs is well established in relation to metastatic NSCLC, the occurrence of immune-related adverse effects in the adjuvant setting, particularly when combined with chemotherapy, remains a significant concern. These adverse events, while generally manageable, can be severe enough to necessitate treatment discontinuation for some patients, and a subset of patients may experience persistent long-term effects. Identifying patients at higher risk for irAEs and developing effective strategies for managing these events will be essential as the use of ICIs expands into earlier stages of the disease.

Additionally, as more immunotherapy agents are approved for NSCLC, it will be critical to identify the most effective combinations of ICIs with other therapeutic modalities, such as chemotherapy. While current evidence supports combining ICIs with chemotherapy, data on the potential synergistic effects of immunotherapy, especially in the adjuvant setting, remain limited. Future studies should investigate how these combinations can be optimized to maximize clinical benefits while minimizing toxicity.

### Limitations and Future Perspectives

Despite the encouraging outcomes observed in recent trials investigating adjuvant immunotherapy for resectable NSCLC, several limitations must be acknowledged when interpreting the current available evidence.

First, most available data derive from phase III clinical trials, which, while methodologically robust, often employ strict inclusion and exclusion criteria that limit the generalizability of findings to real-world populations.

Additionally, the majority of the available data comes from studies involving ICIs in combination with chemotherapy, with limited exploration of other potential combinations or monotherapies, especially in specific subgroups, such as those with a squamous histology or without PD-L1 expression. The heterogeneity of the treatment regimens, particularly the variable use of chemotherapy in the trials (being mandatory in some and optional in others), introduces potential confounding factors that make it challenging to draw definitive conclusions about the independent effect of immunotherapy.

Although adjuvant immunotherapy has demonstrated significant improvements in DFS across several trials, the relatively short follow-up durations limit our understanding of its long-term impact. While encouraging, DFS gains alone are insufficient to determine whether these therapies translate into OS benefits or reduced long-term recurrence. The risk of late recurrences or distant metastases, which may emerge years after treatment, underscores the need for prolonged follow-ups in order to accurately assess both efficacy and safety over time.

Several key trials, including PEARLS/KEYNOTE-091 and IMpower010, have reported notable DFS improvements; however, variations in study design, treatment protocols, and patient populations complicate direct comparisons and the interpretation of results. Other trials such as ACCIO, BR31, and MERMAID-1 also focus primarily on DFS, reinforcing this metric’s relevance as an early efficacy endpoint. Nevertheless, without extended follow-ups, it remains uncertain whether DFS benefits will ultimately translate into durable OS advantages or increased cure rates. Long-term data are therefore essential to fully define the role of adjuvant ICIs in resected cancer settings.

Furthermore, while emerging biomarkers such as ctDNA and TMB are discussed for their potential roles in treatment stratification, their clinical utility is not yet validated. The lack of standardized assays, thresholds, and prospective evidence limits their current applicability in guiding therapy decisions.

Overall, while the integration of ICIs into the adjuvant treatment of resectable NSCLC represents a major advancement, numerous questions remain regarding the optimal use of these therapies. Refining patient selection through the use of multiple biomarkers, determining the ideal duration of treatment, and addressing the management of irAEs will be essential to fully realize the potential of adjuvant immunotherapy in NSCLC. As research progresses, a more personalized approach to treatment will likely emerge, enhancing the effectiveness of immunotherapy and improving survival outcomes for patients with resectable NSCLC.

## 5. Conclusions

These trials are essential for clarifying the efficacy of adjuvant immunotherapy in early-stage NSCLC, with a primary focus on improving survival outcomes and reducing recurrence rates. The exploration of combination therapies and novel immunotherapeutic agents holds significant promise in terms of enhancing the overall treatment landscape. Nevertheless, several limitations should be acknowledged. A number of studies in this area are still ongoing, and for some of the completed trials, mature overall survival data are not yet available, limiting our ability to fully assess long-term outcomes. Additionally, while we selected the most relevant and influential trials, the heterogeneity of study designs, PD-L1 expression thresholds, and patient populations makes direct comparison challenging. The results from these studies are expected to refine current therapeutic protocols, facilitate the identification of optimal patient populations for adjuvant immunotherapy, and ultimately improve long-term prognosis. Further research, particularly on biomarker-driven strategies, will be essential to optimize the integration of adjuvant immunotherapy within personalized treatment regimens.
